# Medical Uses of Electricity

**Published:** 1860-08

**Authors:** 


### BOOK AND PAMPHLET NOTICES.

MEDICAL USES OF ELECTRICITY. By ALFRED GARRATT, M. D.

In a former number of the *Examiner* we barely had time to announce the issue of this work from the press of the enterprising publishers, Ticknor & Fields, Boston.

From a careful examination of its contents we are confident that the medical profession will be under lasting obligation to Dr. Garratt, for a collation of facts not before in accessible form for profitable investigation, for the happy arrangement of his subjects, and the successful manner of their treatment.

The author very justly remarks that "a systematic work on the medical and surgical uses of electricity, containing clear and practical directions as to *where*, *when*, *and how* to employ electricity as a remedy, has long been greatly needed." Many eminent men had requested him to publish the results of his labors in this department of medical science, and the result fully justifies their request, and compliments their judgment. The work is both scientific and practical, a book which should be in the hands of every medical student, and no medical library will be complete hereafter without it.

The author says: "I was fully aware that my position, my views, and my aims might excite misapprehension, because the hitherto very general association of the empirical uses of electricity with quackery, throughout the length and breadth of our country, would naturally lead to some erroneous verdict, at least until my true position might be directly and definitely defined. On the one hand, in regular practice, no surgeon, no oculist, no spinal, or uterine, or urinary doctor; no orthopedist, or general practitioner, shall imagine that I wish to interfere with their respective positions, for which they have especially studied, and in which they are devoting their lives. On the other hand, let no wandering arab of a boasting and quackish 'electro-pathist,' electro-physiologist, or traveling '*galvanizer*,' attempt to screen himself by using my name and address, under any connivance or sympathy."

"Our art is *one* art. Each branch is but a part of the whole, and simply '*E pluribus unum.*' It is too late to be sticklers for creeds or isms, for pathies or systems; only let each be honest and earnest in his professional sphere. The author is desirous that this should no longer be termed a '*system*' of practice, but merely the *electric remedies*, etc., and that we take especial pains to eradicate these false notions from the minds of the people."

Chapter I, describes *natural* electricity, its character, sources, and discovery; presents the theories of Franklin, Symner, and De la Reve; treats of clouds, thunder storms, fogs, relation of electricity to the air and earth; the difference between electricity and magnetism; lightning, and safety from lightning: effects upon the human organism, as regards births and deaths, and of electric changes as the cause of epidemic disease.

Chapter II, presents us with the "Early History of the Medical Uses of Electricity."

Chapter III, treats of "Electrical Instruments and Apparatus for medical purposes," with ample references to the discoveries of Prof. Oersted, Ampere, Sir H. Davy, M. Nobili, Profs. Henry, Faraday, Neef, etc.

Chapter IV. Discusses the subject of Electro-physiology, with citations from Matteucci, Alfred Smee, Becquerel, Marshall Hall, Brown Sequard and others. A chapter of exceeding interest, and to which we shall have occasion again to refer.

Chapter V. Describes the methods for the medical employment of Electricity.

Chapter VI. Discusses Hyperœsthesia.

Chapter VII. Anæsthesia.

Chapter VIII. Spastic Diseases. Views of Marshall Hall on Spinal Diseases, with the researches of Wm. Flourens, Weber, Todd, Hall, Brown, etc.

Chapter IX, has reference to Electro-Therapeutics in Midwifery. Effects of Electricity upon the abdominal viscera, and upon the secretions.

Chapter X. Electricity in Surgery. Its agency in the treatment of "Nervous affections of the Eye, the Ear, Indolent Ulcers, Aneurisms, Ununited Fractures of Bones—as a Moxa, a Cautery, etc.; closing with an article on Surgical Dentistry.

If future trial shall verify the experience and anticipations of Dr. Garratt, we shall be greatly indebted to him for bringing forward these additional means for alleviating misery and curing disease. If it shall not in all respects realize our hopes, still the work will be eminently valuable, as stimulating to investigation in the right direction, and will hasten the exploration of a field of study and practice far too long neglected.

The work will repay an attentive perusal, its illustrations are admirable, and its mechanical execution in all respects a credit to the publishers. J. H. H.

